# Supplementary material for: HAPPY MAMA Project (PART 1). Assessing the Reliability of the Italian Karitane Parenting Confidence Scale (KPCS-IT) and Parental Stress Scale (PSS-IT): A Cross-Sectional Study among Mothers Who Gave Birth in the Last 12 Months
Source: Int J Environ Res Public Health. 2021 Apr 12;18(8):4066. doi: 10.3390/ijerph18084066 (PMC8070573; doi:10.3390/ijerph18084066)
Supplement: Supplementary file 1 [file ijerph-18-04066-s001.pdf]

International Journal of
Environmental Research
and Public Health

MDPI

Supplementary Materials

Annex 1—List of the Facebook groups involved in the study: name and followers in round brackets at May–June 2019.

1. Mamme di Pescara (3000)
2. •La vita da mamma• (1830)
3. 1000mamme (103)
4. donne e Mamme sarde (1000)
5. Franca e i Bebè (2401)
6. gente del lago piemonte e lombardia (3102)
7. MaCs - Mamme A Cosenza (18,399);
8. mamme a Catania (2670)
9. Mamme a Firenze (580)
10. Mamme d'Italia e Non solo (5700)
11. Mamme dell'Emilia Romagna (2173)
12. mamme di Ostia e dintorni (5680)
13. Mamme di Palermo (2985)
14. Mamme di Pescara pubblicità e eventi a go go go (821)
15. Mamme di Pisa e affini (727)
16. Mamme e bambini di Torino (2562)
17. Mamme marsupio di Cagliari (800)
18. mamme.it (262,372)
19. SOLO LE MAMME SANNO (40,299)

20. Stappamamma (3829)

21. universo mamme e non solo (14,000)

22. MAMME GRAVIDANZA!consigli (28,036)

23. Mamme pancine e altro (2287)

24. Tra mamme – Toscana (15,147)

25. Il tempo delle mamme di Ancona e dintorni (14,475)

26. Il club delle MAMME ❤️ (13,048)

27. mamme & pancione (community) (149,812)

28. Le mamme della porta accanto (10,177)

29. Mamme e bimbi 3.0 ❤️ (14,165)

30. Le mamme della porta accanto (10,177)

31. Tra mamme – Toscana (15,147)

32. SOLO LE MAMME SANNO (40,299)

33. Gravidanza, mamme, neo mamme e pancioni (7557)

34. Le Mammole del Reparto Maternità di Cona – Ferrara (1901)

35. Mamme pancine e altro (2287)

36. Mamme e donne del lago Piemonte e Lombardia (3461)

Table S1. The Italian versions of the KPCS and PSS questionnaires.

Items	PSS Original version	PSS-IT
1	I am happy in my role as a parent.	Mi sento felice nel mio ruolo di genitore
2	There is little or nothing I wouldn't do for my child(ren) if it was necessary.	Farei di tutto o quasi per mio/miei figlio/i se fosse necessario
3	Caring for my child(ren) sometimes takes more time and energy than I have to give.	Prendersi cura dei/di miei/mio figli/o a volte richiede più tempo ed energia di quelle che ho da dare

4	I sometimes worry whether I am doing enough for my child(ren).	Mi capita di preoccuparmi di non riuscire a fare abbastanza per mio/miei figlio/i
5	I feel close to my child(ren).	Mi sento vicina a mio/miei figlio/i
6	I enjoy spending time witb my child(ren).	Mi piace trascorrere il tempo con mio/miei figlio/i
7	My child(ren) is (are) an important source of affection for me.	I miei/mio figli/o sono/è un importante fonte di affetto per me
8	Having children gives me a more certain and optimistic view for the future.	Avere figli mi dà una visione più certa e ottimista per il futuro
9	Tue major source of stress in my life is my child(ren).	La più grande fonte di stress nella mia vita sono/è i miei/mio figli/o
10	Having children leaves little time and flexibility in my life.	Avere figli lascia poco tempo e flessibilità nella mia vita
11	Having children bas been a financial burden.	Avere figli è stato un peso economico
12	It is difficult to balance different responsibilities because of my child(ren).	Mi è difficile trovare un equilibrio tra le diverse responsabilità a causa di mio/miei figlio/i
13	Tue behavior of my child(ren) is often embarrassing or stressful to me.	Il comportamento dei miei/mio figli/o è spesso imbarazzante o stressante per me
14	If I had it to do over again, I might decide not to have children.	Se tornassi indietro, potrei decidere di non avere figli
15	I feel overwhelmed by the responsibility of being a parent.	Mi sento sopraffatto dalla responsabilità di essere un genitore
16	Having children bas meant having too few choices and too little control over my life.	Avere figli ha significato troppe poche scelte e troppo poco controllo sulla mia vita
17	I am satisfied as a parent.	Mi sente soddisfatto come genitore
18	I find my child(ren) enjoyable.	Trovo i miei/mio figli/o piacevoli/e

**Items**	**KPCS original version**	**KPCS-IT**
1	I am confident about feeding my baby<br>*Not applicable (my partner feeds the baby)*	Mi sento serena quando do da mangiare al mio bambino<br>*Non applicabile (non do io da mangiare)*
2	I can settle my baby	Mi sento in grado di accudire il mio bambino
3	I am confident about helping my baby to establish a good sleep routine	Mi sento in grado di aiutare il mio bambino a stabilire un buon ritmo del sonno
4	I know what to do when my baby cries	So cosa fare quando il mio bambino piange
5	I understand what my baby is trying to tell mea	Capisco cosa il mio bambino cerca di dire
6	I can sooth my baby when he/she is distressed	Sono in grado di tranquillizzare il mio bambino quando è agitato
7	I am confident about playing with my baby	Mi sento tranquilla quando gioco col suo bambino
8	If my baby has a cold or slight fever, I am confident about handling this	Se il mio bambino ha un raffreddore o una leggera febbre, mi sento sicura nel gestire la situazione

9	I feel sure that my partner will be there for me when I need support<br>Not applicable (I don’t have a partner)	Sono sicura di poter contare sul supporto del mio partner in caso di bisogno. Non applicabile (non ho partner)
10	I am confident that my baby is doing well	Sono sicura che il mio bambino stia bene
11	I can make decisions about the care of my baby	Sono in grado di prendere decisioni riguardo la cura del mio bambino
12	Being a mother is very stressful for me	Essere madre è molto stressante per me
13	I feel I am doing a good job as a mother	Sento che sto facendo un buon lavoro come madre
14	Other people think I am doing a good job as a mother/father	Le altre persone pensano che stia facendo un buon lavoro come madre
15	I feel sure that people will be there for me when I need support	Sono sicura di poter contare sul supporto di altre persone in caso di bisogno
